# Capacity to Elicit Cytotoxic CD8 T Cell Activity Against *Mycobacterium avium* subsp. *paratuberculosis* Is Retained in a Vaccine Candidate 35 kDa Peptide Modified for Expression in Mammalian Cells

**DOI:** 10.3389/fimmu.2019.02859

**Published:** 2019-12-11

**Authors:** Valentina Franceschi, Asmaa H. Mahmoud, Gaber S. Abdellrazeq, Giulia Tebaldi, Francesca Macchi, Luca Russo, Lindsay M. Fry, Mahmoud M. Elnaggar, John P. Bannantine, Kun-Taek Park, Victoria Hulubei, Sandro Cavirani, William C. Davis, Gaetano Donofrio

**Affiliations:** ^1^Department of Medical-Veterinary Science, University of Parma, Parma, Italy; ^2^Department of Veterinary Microbiology and Pathology, Washington State University, Pullman, WA, United States; ^3^Veterinary Quarantine of Alexandria, General Organization for Veterinary Services, Ministry of Agriculture, Alexandria, Egypt; ^4^Department of Microbiology, Faculty of Veterinary Medicine, Alexandria University, Alexandria, Egypt; ^5^Animal Disease Research Unit, Agricultural Research Service (ARS), USDA, Pullman, WA, United States; ^6^National Animal Disease Center, Agricultural Research Service (ARS), USDA, Ames, IA, United States; ^7^Department of Biotechnology, Inje University, Gimhae, South Korea

**Keywords:** Ptb, MMP, mammalian expression vector, immune response, vaccine

## Abstract

Studies focused on development of an attenuated vaccine against *Mycobacterium avium* subsp. *paratuberculosis* (*Map*), the causative agent of paratuberculosis (Ptb) in cattle and other species, revealed that deletion of *relA*, a global gene regulator, abrogates the ability of *Map* to establish a persistent infection. In the absence of *relA*, cattle develop CD8 cytotoxic T cells (CTL) with the ability to kill intracellular bacteria. Analysis of the recall response to a *relA* mutant, *Map*/Δ*relA*, with cells from a vaccinated steer demonstrated that a 35-kDa membrane peptide (MMP) is one of the targets of the response. This observation suggested that it might be possible to develop a peptide-based vaccine. As reported here, the gene encoding the hypothetical MMP ORF, MAP2121c, was modified for expression in mammalian cells as a first step in developing an expression cassette for incorporation into a mammalian expression vector. The modified sequence of MMP, tPA-MMP, was mutated to generate two additional sequences for the study, one with substitutions to replace five potential residues that could be glycosylated, tPA-MMP-5mut, and one with substitutions to replace the first two potential residues that could be glycosylated, tPA-MMP-2mut. The sequences were placed in an expression cassette to produce peptides for analysis. An *ex vivo* platform was used with flow cytometry and a bacterium viability assay to determine if modifications in the gene encoding MMP for expression in mammalian cells altered its capacity to elicit development of CD8 CTL, essential for its use in a peptide-based vaccine. Monocyte-depleted PBMC (mdPBMC) were stimulated with antigen-presenting cells (APC) pulsed with different MMP constructs. CD4 and CD8 T cells proliferated in response to stimulation with MMP (control) expressed in *Escherichia coli* (eMMP), tPA-MMP, and tPA-MMP-2mut. CD8 T cells retained the capacity to kill intracellular bacteria. The tPA-MMP-5mut failed to elicit a proliferative response and was not included in further studies. The data show that the expression cassettes containing MMP and MMP-2mut can be used to screen and select a mammalian expression vector for the development of an efficacious peptide-based vaccine against Ptb.

## Introduction

*Mycobacterium avium* subsp. *paratuberculosis* (*Map*) is a mycobacterial pathogen with a broad host range that includes livestock, humans, and wildlife (1–5). It is the causative agent of paratuberculosis (Ptb) in livestock. Ptb (also referred to as Johne's disease) has become a major disease problem in dairy ruminants, especially in cattle. Similar to other pathogenic mycobacteria, initial infection leads to development of a persistent infection under immune control. For yet unknown reasons, this protective immunity is dysregulated two or more years post-infection, allowing for development of clinical disease. The inability to detect infected animals during the early (latent) stages of infection has led to the inadvertent spread of Ptb via introduction of latently infected animals into clean herds worldwide. Attempts to clear *Map* from livestock herds using management strategies and improved early diagnostic methods has not been successful (6–10). Thus, development of a *Map* livestock vaccine is urgently needed.

Past efforts with killed vaccines revealed that vaccination reduces the incidence of clinical disease, but does not prevent establishment of infection and fecal shedding (11, 12). Similar results have been reported with peptide-based vaccines (13, 14). Subsequently, attenuated *Map* strains were developed as potential live vaccines, and efforts to discover potential peptide vaccine targets continue (13, 15, 16). A major limitation of previous and ongoing endeavors has been the lack of methods to fully evaluate the immune response to candidate live and peptide-based vaccines. As a consequence, there has been a lingering question as to whether there is an age-related difference in susceptibility or immune-responsiveness to *Map* infection that confers long-lasting immunity in adult animals (6). We developed the reagents and methods needed to study the *ex vivo* immune response to *Map* to answer this question, and to analyze the immune response to candidate vaccines.

Initial studies comparing the immune response to *Map* in experimentally and naturally infected cattle revealed a possible age-related difference in the development of the CD8 T cell response to mycobacterial antigens (17, 18). The proliferative response of CD4 T cells to mycobacterial antigens was early and vigorous in experimentally infected calves, and the CD8 T cell response increased over time (18). The CD4 and CD8 T cell proliferative responses to mycobacterial antigens were strong in naturally infected cattle. Although differences in immune responses were detected, no age-related difference in susceptibility to infection was found. Adaptation of methods to use site-directed mutagenesis with *Map* provided an opportunity to develop and compare the immune response to mutants with deletions in genes associated with virulence (19). We were particularly interested in *relA*, a global gene regulator. Studies with a *relA* deletion mutant in *Mycobacterium tuberculosis* (*Mtb*) in a mouse model revealed that the mutant was unable to establish a persistent infection (20).

A *relA* deletion mutant was developed in *Map* (Δ*Map*/*relA*), and a similar decrease in bacterial survival was noted (21). Analysis of the *ex vivo* immune response revealed the loss of ability to establish a persistent infection was attributable to development of CD8 cytotoxic T cells (CTL) with the ability to kill intracellular bacteria, a function essential for development of an attenuated mutant vaccine for *Map* (22). Further analysis of the recall response to Δ*Map*/*relA* using PBMC from a steer vaccinated with the Δ*Map*/*relA* mutant revealed that the target of the response was a 35-kDa membrane-associated molecule encoded by MAP2121c, MMP (22, 23). Comparison of the recall response demonstrated a comparable CTL response could be elicited with live Δ*Map*/*relA* or MMP, suggesting that it might be possible to develop an MMP peptide-based vaccine. *Ex vivo* studies demonstrated that the same CD8 CTL activity could be elicited with antigen-presenting cells (APC) pulsed with MMP alone or incorporated into a nanoparticle-based vector (24). The most recent studies revealed two important findings. The first is that the nanoparticle (NP)-based approach to peptide-based vaccination is not the best for production of a vaccine for field use, and that other methods should be explored. The second finding relevant to development of vaccines in general is that development of CD8 CTL against MMP only occurred if CD4 and CD8 T cells recognized their respective antigenic epitopes, presented by APC, at the same time (submitted for review). The proliferative and CD8 CTL response to antigens presented by APC pulsed with MMP was blocked in the presence of antibody to either MHC class I or class II molecules. This finding revealed that any form of MMP developed for use in a vaccine must be designed in a configuration that retains the features of the native molecule associated with antigen processing for cross-presentation of peptides in the context of MHC class I and class II molecules.

The present study was conducted to determine if the native gene sequence encoding MMP could be modified for expression in mammalian cells and retain its full immunogenic properties, a prerequisite for its potential use in a vector designed for expression in mammalian cells.

## Materials and Methods

### Mammalian Cell Lines

Human Embryo Kidney (HEK) 293T (ATCC: CRL-11268) and Human Kidney/B Cell Hybrid (HKB-11) (ATCC: CRL-12568) cells were maintained as suggested by the manufacturer's instructions. All cell lines were cultured in Eagle's Minimal Essential Medium (EMEM, Gibco) containing 10% fetal bovine serum (FBS), 2 mM of L-glutamine (Gibco), 100 IU/ml of penicillin (Gibco), 100 μg/ml of streptomycin (SIGMA), and 0.25 μg/ml of amphotericin B (Gibco), and were incubated at 37°C/5% CO_2_ in a humidified incubator.

### Sequence Analysis and Cloning of MMP

The human codon-usage adapted MMP synthetic ORF was modified from the published sequence for the hypothetical protein MAP_2121c, *Map* K-10 (NCBI GenBank: AAS04438.1). In this synthetic construct, a Kozak's sequence, the human tissue plasminogen activator signal peptide (tPA), and an AU1 peptide tag were added to the human-adapted ORF sequence at the 5′ terminus and at the 3′ terminus, respectively, generating tPA-MMP. In addition, two synthetic mutants, tPA-MMP-5mut and tPA-MMP-2mut, were generated. In this step, all five predicted N-glycosylation sites (tPA-MMP-5mut) or only the first two predicted N-glycosylation sites (tPA-MMP-2mut) were mutated.

Using PCR amplification, restriction sites were added to the 3′ and 5′ termini of tPA-MMP, tPA-MMP-5mut, and tPA-MMP-2mut to facilitate subsequent cloning. Briefly, the primer pair NheI-p35-sense (5′-CCCCGCTAGCCCACCATGGACGCTATGAAGAGGGGCCTGTGCTGC-3′) and SmaI-p35-antisense (5′-CCCCCCCGGGTTAGATGTACCGGTAGGTGTCCTTGTACTC-3′) was used to insert a NheI restriction site at the 5′ end and a SmaI restriction site at the 3′ end of the ORF. The PCR amplification reaction was performed in a final volume of 50 μl, containing 10 mM Tris–hydrochloride pH 8.3, 10% dimethyl sulfoxide (DMSO), 0.2 mM deoxy nucleotide triphosphates, 2.5 mM MgSO_4_, 50 mM KCl, and 0.25 μM of each primer. tPA-MMP, tPA-MMP-5mut, and tPA-MMP-2mut DNA were first linearized with HindIII to facilitate polymerase action and then amplified over 35 cycles consisting of 1 min of denaturation at 94°C, 1 min of primer annealing at 60°C, and 1 min of chain elongation with 1 U of Pfu DNA polymerase (Thermo Scientific) at 72°C. The amplicons were subsequently checked in 1% agarose gel and visualized using ethidium bromide staining in 1× TAE buffer (40 mM Tris–acetate, 1 mM EDTA).

Later, the NheI/SmaI cut amplicons were cloned into the shuttle vector pINT2EGFP (25) and cut with the same enzymes, generating pCMV-tPA-MMP, pCMV-tPA-MMP-5mut, and pCMV-tPA-MMP-2mut, thereby placing the tPA-MMP ORFs under the transcriptional control of the immediate early gene promoter of human cytomegalovirus (CMV), followed by the bovine growth hormone polyadenylation signal (pA). Alternatively, the NheI/SmaI cut amplicons were subcloned into the vector pEF1α-p67 (26) to put the tPA-MMP ORFs under the transcriptional control of the Human Elongation Factor 1 Alpha (EF1α) promoter followed by the bovine growth hormone pA, thus generating pEF1α-tPA-MMP, pEF1α-tPA-MMP-5mut, and pEF1α-tPA-MMP-2mut.

The pEF1α-tPA-MMP-CMV-EGFP:T2A:Puro lentiviral vector was obtained by subcloning the NheI/SmaI cut, tPA-MMP amplicon into the third-generation, replication-incompetent, lentiviral vector pLV-EGFP:T2A:Puro (Cyagen), linearized using NheI/EcoRV digestion, to place the modified tPA-MMP sequence under the transcriptional control of the EF1α promoter.

### PNGase F Digestion

PNGase F (New England BioLabs) was used as suggested by the manufacturer. Briefly, serum-free medium supernatants from pEF1α-tPA-MMP, pEF1α-tPA-MMP-5mut, and pEF1α-tPA-MMP-2mut-transfected HEK cells, collected after 72 h of secretion, were digested with PNGase F, which cleaves between the innermost N-Acetyl-D-Glucosamine (GlcNAc) and asparagine residues from N-linked glycoproteins. Twenty micrograms of glycoproteins was denatured at 100°C for 10 min in the presence of 10× Glycoprotein Denaturing Buffer; after chilling on ice and a brief centrifuge of 10 s, GlycoBuffer 2 (10×), NP-40 10%, water, and 1 μl of PNGase F (500,000 U/ml) were added. The reaction was incubated for 1 h at 37°C. After PNGase digestion, tPA-MMP was detected using Western immunoblotting as described below.

### Transient Transfection and Secretion of tPA-MMP From Hek 293T Cells

To evaluate the expression and secretion of tPA-MMP, HEK 293T cells were transiently transfected with pCMV-tPA-MMP, pCMV-tPA-MMP-5mut, pCMV-tPA-MMP-2mut, pEF1α-tPA-MMP, pEF1α-tPA-MMP-5mut, pEF1α-tPA-MMP-2mut, pEF1α-tPA-MMP-CMV-EGFP:T2A:Puro, pEF1 α-iresGFP (pWPI, from Addgene), or pEGFP-C1 (Clontech), using polyethylenimine (PEI) transfection reagent (Polysciences, Inc.). Briefly, cells were seeded at 3 × 10^5^ cells/well in six-well plates and incubated overnight at 37°C/5% CO_2_ in a humidified incubator. Cells were then incubated for 6 h with a transfection mix containing 3 μg of plasmid DNA and PEI (ratio 1:2.5 DNA-PEI) in Dulbecco's modified essential medium (DMEM) high glucose (Euroclone) without serum. After incubation, the transfection mix was replaced by fresh medium EMEM, with 10% FBS, 100 IU/ml of penicillin, 100 μg/ml of streptomycin, and 0.25 μg/ml of amphotericin B, and incubated for 24 h at 37°C/5% CO_2_ in a humidified incubator. To test supernatant protein expression, the transfection solution was replaced with fresh DMEM/F12 (ratio 1:1) medium without FBS and incubated for 48 h at 37°C/5% CO_2_ in a humidified incubator. Cell supernatant was then collected and analyzed by immunoblot.

### Lentivirus Reconstitution and Transduction

HEK 293T cells were transfected in a T175 cm^2^ flask with 25 μg of pEF1α-tPA_MMP-CMV-EGFP:T2A:Puro or pEF1α-iresGFP transfer vectors, 13 μg of p8.74 packaging vector, 10 μg of pMD2 pseudotyping vector, and 10 μg of pREV using PEI transfection reagent (Polysciences, Inc.). Briefly, 58 μg of DNA was mixed with 145 μg of PEI (1 mg/ml) (ratio 1:2.5 DNA-PEI) in 3 ml of Dulbecco's modified essential medium (DMEM) high glucose (Euroclone) without serum. After 15 min of incubation at room temperature, 4× volumes of medium without serum were added and the transfection solution was transferred to the cell monolayer and left for 6 h at 37°C/5% CO_2_, in a humidified incubator. The transfection mixture was then replaced with 25 ml of fresh medium EMEM supplemented with 10% FBS, 50 IU/ml of penicillin, 50 μg/ml of streptomycin, and 2.5 μg/ml of amphotericin B, and cells incubated for 48 h at 37°C/5% CO_2_. The flask was then stored at −80°C. Lentivirus was later obtained by subjecting cells to three freeze–thaw cycles and then clarifying the supernatant via centrifugation at 3500 rpm for 5 min at 4°C and filtering through a 0.45-μm filter (Millipore). The clarified, lentivirus-containing supernatant was stored at −80°C. To obtain stably transduced cell lines, 1 × 10^5^ HKB-11 cells were infected with 2 × 10^5^ T.U. (transducing units) of reconstituted tPA-MMP lentivirus. Cells were incubated overnight at 37°C/5% CO_2._ The culture medium was then replaced with fresh medium supplemented with 10% of FBS. Transduced cells were observed daily via fluorescence microscopy for green fluorescence protein (GFP) expression to monitor the rate of transduction and were selected with 1 μg/ml of Puromycin to achieve a 100% transduction rate.

### Immunoblot

Protein cell extracts were obtained from pCMV-tPA-MMP, pCMV-tPA-MMP-5mut, pCMV-tPA-MMP-2mut, pEF1α-tPA-MMP, pEF1α-tPA-MMP-5mut, pEF1α-tPA-MMP-2mut, and pEGFP-C1 transfected HEK 293T, or from tPA-MMP lentivirus stably transduced HKB-11 cells and non-transduced control HKB-11 cells by adding 100 μl of cell extraction buffer (50 mM Tris-HCl, 150 mM NaCl, and 1% NP-40; pH 8). After BCA total protein quantification (Pierce™ BCA Protein Assay kit, Thermo Scientific), cell extracts containing various amount of total protein were electrophoresed through 10% SDS-PAGE. Proteins were then transferred to nylon membranes by electroblotting, and membranes were incubated with anti-AU1 rabbit polyclonal antibody (A190-125A, Bethyl Laboratories Inc.) diluted 1:10.000, washed, and then incubated with a goat anti-rabbit secondary antibody labeled with horseradish peroxidase (Sigma), diluted 1:15,000 and visualized by enhanced chemi-luminescence (Clarity Max western ECL substrate, Biorad). Also, cell supernatants, obtained from HEK 293T cells transfected with pCMV-tPA-MMP, pCMV-tPA-MMP-5mut, pCMV-tPA-MMP-2mut, pEF1α-tPA-MMP, pEF1α-tPA-MMP-5mut, pEF1α-tPA-MMP-2mut, and pEGFP-C1 and from all the transduced cell lines, were collected after 72 h in serum-free medium DMEM-F12 secretion condition and analyzed through 10% SDS–PAGE gel electrophoresis.

### Blood Source

Blood required to conduct the study of the immune response to mammalian-expressed MMP peptides was obtained from three *Map*-free Holstein steers housed in an open feed lot and maintained by the WSU animal care staff. All protocols were approved by the Washington State University Institutional Animal Care and Use Committee (ASAFs 3360 and 04883).

### Cell Separation and Culture

Peripheral blood mononuclear cells (PBMC) were prepared by density gradient centrifugation. One portion of the PBMC was used to generate monocyte-derived macrophages (MoMΦ) for use in a propidium monoazide quantitative PCR (PMA-QPCR) viability assay (22). This portion of the PBMC was re-suspended in complete RPMI culture medium (cRPMI) [RPMI-1640 medium with GlutaMAX^TM^ (Life Technologies, CA) supplemented with 10% calf bovine serum (CBS), 1 mM β-mercaptoethanol, 100 units/ml of penicillin G, and 100 μg/ml of streptomycin sulfate] and cultured overnight in 150-mm tissue culture plates. The following day, non-adherent cells were removed and adherent cells were cultured in fresh medium for 6 days. On day six, the adherent cells were brought into suspension by incubation on ice in the presence of ethylenediaminetetraacetic acid (EDTA) in phosphate buffered saline (PBS) [4 ml EDTA (250 mM stock in H_2_O), 5 ml CBS, and 91 ml PBS]. Then, the MoMΦ were distributed into six-well culture plates (2 × 10^6^ cells/well) and cultured for an additional 6 days in fresh cRPMI at 37°C/5% CO_2_.

The second PBMC portion was subjected to magnetic separation using microbeads coated with a cross-reactive anti-human CD14 mAb to isolate monocytes per the manufacturer's instructions (Miltenyi Biotec) (27). Isolated monocytes (2 × 10^6^) were added to wells of six-well culture plates and cultured in 3 ml of cRPMI in the presence of a DC growth cocktail containing bovine GM-CSF and IL-4 according to the manufacturer instructions (Kingfisher Biotech, MN). On the third day, 1.4 ml of the medium was replaced with 1.8 ml of fresh medium containing the cocktail. The cultures were incubated for an additional 3 days to obtain monocyte-derived dendritic cells (MoDC).

Flow-through containing monocyte-depleted PBMC (mdPBMC) were subjected to two rounds of antigenic stimulation using APC (cDC present in mdPBMC) (22, 28) pulsed with MMP expressed in *E. coli* (eMMP) (29) or tPA-MMP, tPA-MMP-5mut, or tPA-MMP-2mut. To conduct the first round of stimulation, mdPBMC were distributed in the six-well culture plate in duplicate (2 × 10^6^/ml in 5 ml of cRPMI). Peptides were added to the indicated wells in a concentration of 5 μg/ml, and the cultures incubated for 6 days at 37°C, 5% CO_2_ to allow antigen processing and presentation by conventional dendritic cells (cDC) present in the mdPBMC. To conduct the second round of stimulation after 6 days of culture, peptides were added to the cultures of MoDC at the same concentration and incubated for 3 h at 37°C, 5% CO_2_. Then, peptide-pulsed MoDC were washed three times with warm RPMI to remove cell-free antigens. The non-adherent primed PBMC were collected, washed with warm RPMI, and then added to their respective, autologous, antigen-pulsed MoDC (2 × 10^6^/ml in 5 ml of cRPMI). After an additional 6 days of culture, the cells were collected and used in FC and the *Map* PMA-QPCR viability assay as described below. One set of mdPBMC were maintained as negative controls with no antigen stimulation during the 2 weeks of cell culture.

### Flow Cytometry

Cell activation and proliferation were assessed using FC as previously described (22). Briefly, cells were washed once in PBS/ACD, re-suspended in serum-free RPMI, and counted. Cells were distributed into 96-well polystyrene V-bottom microplates (10^6^ cells/well) and labeled with combinations of mAbs obtained from the WSU Monoclonal Antibody Center (WSUMAC) (Table 1).

**Table 1:** mAbs used in the present study.

**mAb clone**	**Isotype**	**Specificity/source**	**Fluorochrome**
CAM36A	IgG1	CD14 WSUMAC	Alexa Fluor®647
209MD26A	IgG2a	CD209 WSUMAC	PE CY5.5
ILA11A	IgG2a	CD4 WSUMAC	PE CY5.5
CACT138A	IgG1	CD4 WSUMAC	Alexa Fluor®647
7C2B	IgG2a	CD8 WSUMAC	PE CY5.5
CACT80C	IgG1	CD8 WSUMAC	Alexa Fluor®647
ILA116A	IgG3	CD45R0 WSUMAC	Alexa Fluor®488; PE CY7
CACT116A	IgG1	CD25 WSUMAC	Alexa Fluor®647

*Data were collected on a modified FACS Calibur DxP8 Analyzer equipped with a FlowJo operating system (Cytek Biosciences Inc., Fremont, CA) and analyzed with FCS Express software (DeNovo Software, Glendale, CA) (30). For data acquisition, side scatter (SSC) and forward scatter (FSC) were used to identify small and large lymphocytes. FSC-Height vs. FSC-Width (FSC-H vs. FSC-W) was used to exclude doublets. Additional gates were used to select CD4 and CD8 T cells for determination of their activation status and proliferation*.

### PMA-QPCR Viability Assay

To determine the cytotoxic activity of effector T cells generated after two rounds of stimulation with the different peptides, the live *Map* burden in infected MoMΦ co-cultured with the effector T cells was assessed using the PMA-QPCR viability assay. In this case, one aliquot of *Map* K10 stock (ATCC BAA-968/K10) was inoculated in Middlebrook 7H9 broth (Difco, BD Biosciences, USA) supplemented with 6.7% para-JEM GS (Trek Diagnostic Systems, OH), 2 μg/ml mycobactin J (Allied Monitor, MO, USA), and 0.05% Tween 80 (Sigma-Aldrich Corp.) (19, 21). The cultures were expanded on a shaking stand at 37°C until they reached an OD_600_. Master stock for immediate use in each experiment was prepared in 1.5 ml micro-centrifuge screw-cap tubes. Bacterial suspension was disaggregated by passages through a 26-gauge needle three times, and bacterial numbers were estimated based on the final OD_600_ values (19) and used for MoMΦ infection.

MoMΦ were infected with *Map* K10 at a multiplicity of infection (MOI) of 10:1 (2 × 10^7^ *Map* to ~2 × 10^6^ MoMΦ/well) in antibiotic-free cRPMI for 3 h as previously described (22). Antigen stimulated and control unstimulated mdPBMC were collected and co-cultured with the infected MoMΦ for 24 h at 37°C, 5% CO_2_. All non-adherent and adherent cells per well were collected and lysed for 15 min using 0.01% saponin in H_2_O at 37°C. A set of controls (100% live, 50% live/50% killed, and 100% killed) was prepared from known mixtures of live and dead *Map* K10 to cover the dynamic range of Cycle threshold (C_T_) values for live vs. dead *Map* obtained from infected MoMΦ before and after incubation with the effector cells. Each aliquot contained 2 × 10^7^ total *Map*. Aliquots were used to infect MoMΦ cultures as described followed by co-culture with 10^7^ fresh mdPBMC per well. Then, all cells in each well were immediately lysed with saponin as described above. All lysates were then treated with propidium monoazide (PMA) in a final dye concentration of 50 μM as previously described (22) followed by light exposure for 5 min using a halogen lamp with a 650 W bulb. Cells were subsequently processed for DNA isolation according to the protocol for Gram-positive bacteria using DNeasy® Blood and Tissue kit (Qiagen, USA) as described by Park et al. (31).

The single-copy F57 gene specific for *Map* was used in the TaqMan Quantitative Real-Time PCR (F57 qRT-PCR) to determine the viability of intracellular *Map* as described by Kralik et al. (32) and Abdellrazeq et al. (22). Pure *Map* gDNA was used to generate a standard curve using eight dilutions starting with 4 × 10^7^ copies. The reaction was performed according to Schönenbrücher et al. (33) using a StepOnePlus Real-Time PCR machine (Applied Biosystems, CA). The mean values of the C_T_ were analyzed using StepOne Software v2.1 (Applied Biosystems, CA).

### Statistics

Data were imported into SAS software (SAS for Windows version 9.3 and JMP version 12.0.1; SAS Institute Inc., Cary, NC) for statistical analysis and graphical presentation (means and standard deviations). Two-way ANOVA was used to conduct statistical analyses. *Post-hoc* multiple comparisons were conducted using Tukey HSD (overall α = 0.05). ^*^P < 0.05; ^**^P < 0.01; ^***^P < 0.001.

## Results

### Topological Prediction

Initial data obtained with MMP demonstrated that it is a good candidate antigen for use in a peptide-based vaccine (22). MMP incorporated into an NP vector retained its CTL activity (24). However, methods for incorporating the peptide into the NP vector proved unreliable with some preparations having no activity. Therefore, we explored the potential of modifying the gene encoding MMP for expression in a mammalian expression vector for use in cattle and other species. It was unclear whether modifications in the amino acid sequence needed for expression in mammalian cells would alter the immunogenicity of MMP. Initial studies suggested that MMP is membrane associated (29). Further studies revealed that the peptide is associated with a cysteine desulferase (MAP2120c) in the membrane (23). In the present study, *in silico* analysis using the Phobius (http://phobius.sbc.su.se/) prediction server for presence of transmembrane domains and signal peptides did not detect a canonical signal peptide or hydrophobic region (Supplementary Figure 1). Indeed, an MMP-like sequence in mammalian cells appeared to be intracellularly localized and very soluble.

### Codon Usage Adaptation and Synthetic Gene Design

**Figure 1:**
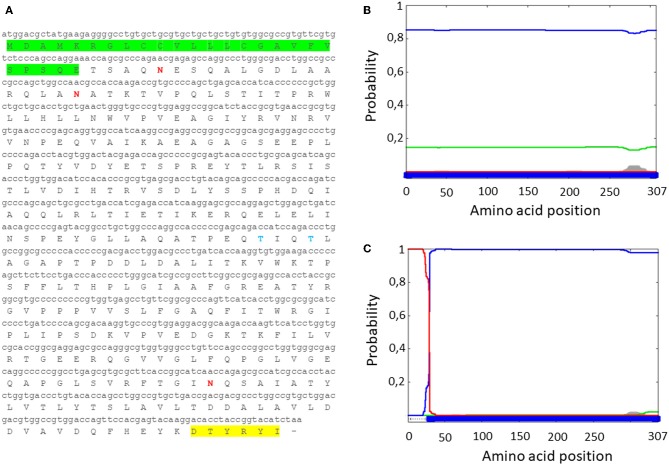
**(A)** MMP nucleotide sequence with human tPA signal peptide (green), along with the deduced amino acid sequence (tPA-MMP). The AU1 tag is highlighted in yellow. **(B)** Server output prediction of transmembrane topology and signal peptides from the amino acid sequence of MMP protein compared with **(C)** MMP amino acid sequence with the human tPA signal peptide (tPA-MMP). In tPA-MMP **(C)**, but not MMP **(B)**, a signal peptide is detected and the protein is predicted to be secreted, reaching the top score of probability (1).

Since MMP is a bacterial protein, codon usage had to be modified before attempting to develop an expression cassette for use in mammalian cells. This was accomplished with use of the Jcat codon adaptation tool to change the MMP ORF nucleotide codon composition to a composition based on human genome codon usage (http://www.jcat.de/) (Supplementary Figure 2). A Kozak's sequence and a human tissue plasminogen activator (tPA) signal peptide were added to the modified MMP to generate tPA-MMP, followed by addition of two restriction enzyme sites to both ends to improve the translation, secretion, and subcloning into a suitable vector. The modified tPA-MMP was sent out for synthesis. Reanalysis of the modified sequence using Phobius revealed that the addition of the signal peptide to generate the tPA-MMP ORF customized for human codon usage shifted its predicted sub-cellular localization to a secreted protein (Figure 1).

### Construction and Transient Expression of tPA-MMP in a Secreted Form

**Figure 2:**
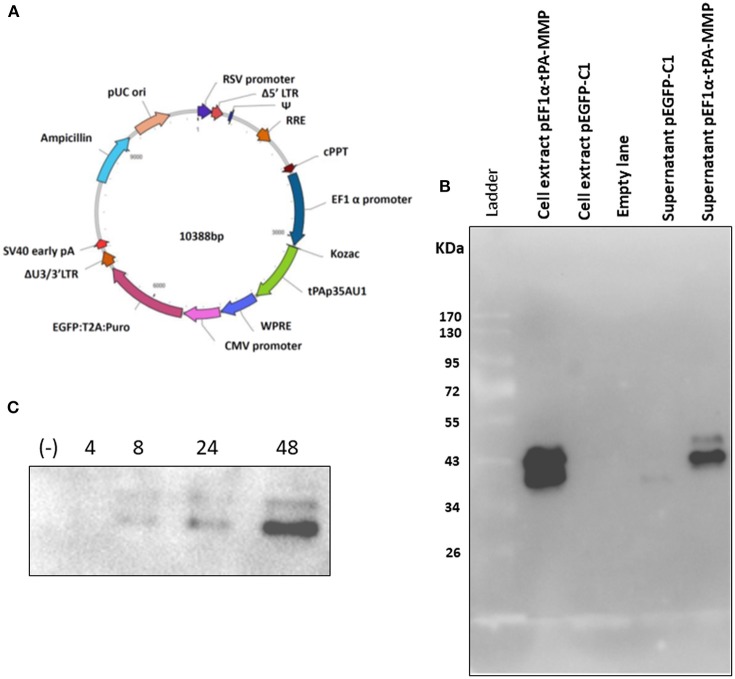
**(A)** Diagram of a third-generation lentiviral transfer vector delivering tPA-MMP ORF (tPAp35AU1, green) expression cassette and GFP/Puromycin reporter gene/selectable marker (EGFP:T2A:Puro, purple) expression cassette. Different genetic elements depicted within the construct from nucleotide position 0 are: RSV promoter (blue violet); Δ5′ LTR (brown); Ψ (medium blue); RRE (chocolate); cPPT (brown); EF1α (navy); WPRE (royal blue); CMV promoter (orchid); ΔU3/3' LTR (sienna); Sv40 early pA (red); Ampicillin (turquoise); pUC ori (dark salmon). **(B)** Western immunoblot of pEF1α-tPA-MMP and pEGFP-C1 transfected HEK 293T cell extracts and supernatant. Lanes with cell extract were loaded with 20 μg of total protein, whereas the lanes with supernatant were loaded with 20 μl of serum-free medium incubated for 48 h with transfected cells. Cells transfected with pEGFPC-1 served as negative controls (*Mock*). **(C)** Western immunoblot of pEF1a-tPA-MMP-CMV-EGFP:T2A:Puro-transduced and Puromicin-selected HKB-11 cells, supernatant collected at 4, 8, 24, and 48 h incubation. In each lane, 15 μl of conditioned serum-free medium was loaded. The negative control (–) was established with a similar lentiviral vector delivering only GFP (pEF1α-iresGFP).

As described, the tPA-MMP was provided with a heterologous signal peptide to facilitate expression and secretion by mammalian cells. The synthetic tPA-MMP (Figure 1A) was placed under the transcriptional control of the Elongation Factor 1 alpha (EF1α) promoter, followed by the bovine growth hormone polyadenylation signal to obtain an EF1α-tPA-MMP expression cassette. The expression cassette was integrated into a mammalian expression vector (pEF1α-tPA-MMP) and into a lentiviral vector (pEF1α-tPA-MMP-CMV-EGFP:T2A:Puro) (Figure 2A). pEF1α-tPA-MMP transiently transfected HEK293 cells and pEF1α-tPA-MMP-CMV-EGFP:T2A:Puro transduced HEK 293T cells secreted the tPA-MMP peptide (Figures 2B,C).

### tPA-MMP Peptide Expressed by Mammalian Cells Is Glycosylated

**Figure 3:**
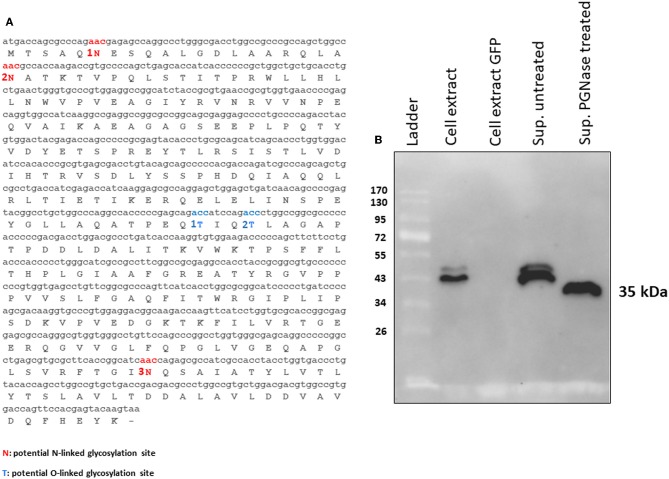
**(A)** MMP N-linked (1N, 2N, and 3N in red) and O-linked (1T and 2T in blue) predicted glycosylation sites. **(B)** Western immunoblot of tPA-MMP secreted protein treated (Sup PNGase treated) or untreated (Sup untreated) with PGNase F. Cell extracts from pEF1α-tPA-MMP (Cell extract) and pEGFP-C1 (Cell extract GFP) transfected HEK 293T cells were used as positive and negative controls, respectively.

**Figure 4:**
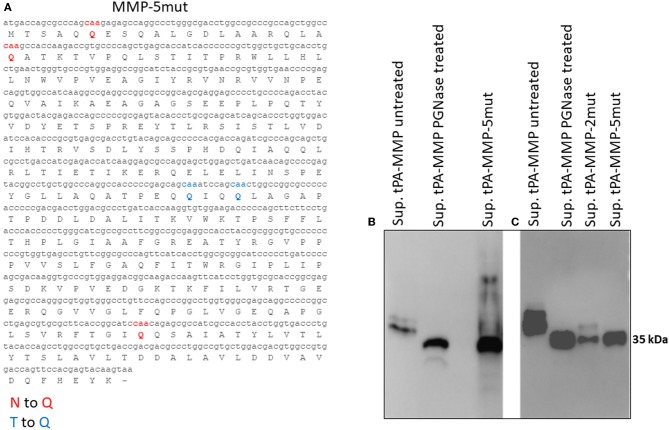
**(A)** Mutated MMP protein sequence (tPA-MMP-5mut), where the predicted N-linked asparagine residues were substituted with glutamine (Q, red) and the predicted O-linked threonine residues were also substituted with glutamine (Q, blue). **(B)** Western immunoblot of secreted tPA-MMP-5mut (Sup tPA-MMP-5mut) compared with tPA-MMP treated or untreated with PNGase. tPA-MMP-5mut and tPA-MMP treated with PNGase exhibit the same electrophoretic mobility. **(C)** Western immunoblot of secreted tPA-MMP-5mut (Sup tPA-MMP-5mut) compared with tPA-MMP-2mut (Sup tPA-MMP-5mut) and tPA-MMP treated or untreated with PNGase. tPA-MMP-5mut, tPA-MMP-2mut, and tPA-MMP treated with PNGase exhibit the same electrophoretic mobility.

tPA-MMP products extracted and secreted from mammalian cells migrated with a lower mobility than expected (Figure 2B), indicating a molecular weight higher than 35 kDa (~43 kDa), which is in contrast with what was previously published (34). In fact, MMP was originally called the “*M. paratuberculosis* 35 kDa major membrane protein” (34). Based on this observation, a possible glycosylation of MMP expressed in mammalian cells was considered. Moreover, tPA-MMP detected in the cell extract had two bands: One could be the glycosylated fraction, and the second, the un-glycosylated fraction of the protein (Figure 2B). The tPA-MMP detected in the secreted peptide fraction showed just a single band of ~43 kDa, attributable to a glycosylated protein form of MMP, in agreement with the fact that secreted proteins in mammalian cells are post-translationally modified, in this case, glycosylated. To explore this possibility, the MMP peptide-modified sequence was analyzed with different glycosylation site prediction programs (Glyco EP, http://www.imtech.res.in/cgibin/glycoep/glyechk?job=932&tim=45; NetGlyc 1.0, http://www.cbs.dtu.dk/services/NetNGlyc/; Protter, http://wlab.ethz.ch/protter/#). All three of the analysis programs yielded highly concordant results. Three potential N-linked and two O-linked glycosylation sites were predicted (Figure 3A). Among all these predicted sites, the first two N-linked glycosylation sites showed the higher scores in terms of prediction. This prediction was validated by treating the secreted tPA-MMP with PNGase F, an amidase that cleaves between the innermost GlcNAc and asparagine residues of high mannose. As shown in Figure 3B, PNGase F treatment of tPA-MMP shifted its molecular size down to ~35 kDa. These data were further corroborated by mutating the five potentially glycosylated residues of tPA-MMP (tPA-MMP-5mut), where the three asparagines and the two threonines were substituted with glutamine in order to eliminate all potential glycosylation sites (Figure 4A). Secreted tPA-MMP-5mut showed the same mobility of secreted, PNGase F-treated tPA-MMP (Figure 4B). Therefore, it was hypothesized that there is no O-linked glycosylation and that tPA-MMP N-linked glycosylation is only related to the first two asparagine residues, affirming the higher prediction scores of the first two predicted N-linked glycosylation sites in respect to the third predicted site. This hypothesis was further validated by generating a second tPA-MMP mutant, tPA-MMP-2mut, in which only the first two asparagine residues were substituted with glutamine (Supplementary Figure 3). In fact, secreted tPA-MMP-2mut, tPA-MMP-5mut, and PNGase F-treated tPA-MMP showed identical patterns of electrophoretic mobility (Figure 4C).

### Expression Levels of tPA-MMP-2mut and tPA-MMP-5mut Are Lower Than that of tPA-MMP

**Figure 5:**
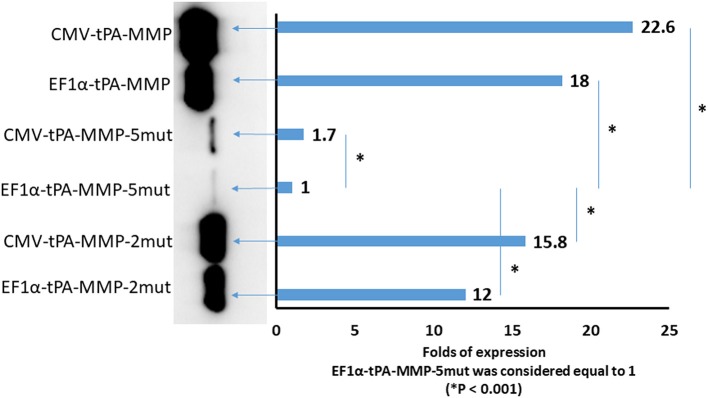
Quantitative Western immunoblot of serum-free medium containing secreted tPA-MMP-5mut, tPA-MMP-2mut, and tPA-MMP from pCMV-tPA-MMP-5mut, pEF1α-tPA-MMP-5mut, pCMV-tPA-MMP-2mut, pEF1α-tPA-MMP-2mut, pCMV-tPA-MMP, and pEF1α-tPA-MMP transfected HEK 293T cells at 48 h post transfection. Each lane was loaded with 15 μl of serum-free medium supernatant. The same number of HEK 293T cells were transfected with the same amount of each DNA plasmid with the same efficiency of transfection (100%), as determined by co-transfection with pEGFP-C1. Band intensity was quantified using densitometry (ChemiDoc; MPIMAGING SYSTEM, LAB SOFTWARE, BioRad), and displayed using a bar graph. Data were normalized as folds of increased secretion, where pEF1α-tPA-MMP-5mut signal was considered equal to 1. The experiment was repeated three times, and statistical significance (*P* < 0.001) was assessed using a Student's *t*-test and one-way ANOVA.

Although tPA-MMP-2mut, tPA-MMP-5mut, and tPA-MMP were expressed, quantitative differences were observed in the amount of secreted product. Therefore, all three constructs were cloned under the control of two different promoters, EF1α and CMV, to exclude a different promoter contribution in terms of transcription. Intracellular protein production was analyzed to assess the impact of protein glycosylation on protein secretion. As shown in Figure 5, less of the tPA-MMP-5mut of the intracellularly expressed and secreted products were expressed in comparison with tPA-MMP-2mut and tPA-MMP expressed and secreted products. Since the tPA-MMP-5mut is un-glycosylated like tPA-MMP-2mut, and the tPA-MMP has similar intracellular expression and secretion levels compared to tPA-MMP-2mut, the quantitative expression difference could be attributed to tPA-MMP-5mut ORF mutations that might affect tPA-MMP-5mut transcription stability. Studies were conducted to explore this possibility. Similar data were obtained using the two different transcription promoters on the three different tPA-MMP ORFs (Figure 5), thus excluding transcriptional differences as an explanation for the observed differences in the level of expression of products. Expression appeared to be slightly more efficient under the control of the CMV promoter in the transient transfection assay.

### Comparison of the CD4 and CD8 T Cell Proliferative Response to APC Pulsed With eMMP, tPA-MMP, tPA-MMP-2mut, tPA-MMP-5mut Peptides-Stimulated Cells

Development of an MMP expression cassette for insertion into a mammalian expression vectors was successful. The next series of studies were conducted to establish that the modified expressed products retained the immunogenic and functional activities of the native peptide expressed in *E. coli*, eMMP. The *ex vivo* platform developed to study the immune response to candidate vaccine antigens was used in conjunction with a bacterium viability assay to compare the immune response of the modified tPA-MMP products with the immune response elicited with eMMP (22). The initial studies conducted during the development of the assays revealed that the flow cytometric assay could be used to phenotype the lymphocytes proliferating in response to stimulation with APC [conventional dendritic cells (DC) present in PBMC depleted of monocytes and monocyte derived DC] pulsed with live bacteria or peptides. For study of the recall and CTL response, one round of stimulation with antigen was sufficient to elicit a proliferative response for phenotyping following 6 days of culture and for analysis of their functional activity using the bacterium viability assay. Two rounds of stimulation with APC pulsed with antigen were needed to expand the culture of proliferating antigen specific T cells for flow cytometry and for analysis of their functional activity using the bacterium viability assay. This time interval provided sufficient time to generate autologous monocyte-derived macrophages for use as infected target cells. As outlined in section Materials and Methods, half the respective preparations of control and stimulated cells were used for phenotyping, and the other half were used in the killing assay. The latter cell preparations were added to the cultures of autologous infected macrophages for 24 h. Then, all the cells in the respective cultures were collected and lysed to isolate bacterial DNA for viability analysis.

### Flow Cytometric Analysis of the Proliferative Response

**Figure 6:**
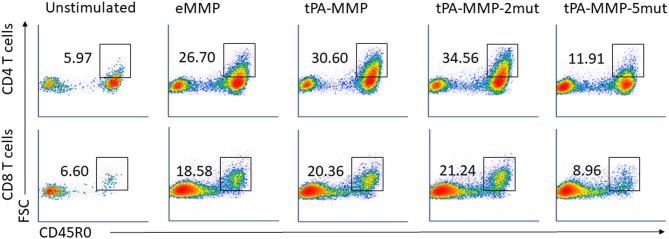
Representative FC profiles comparing CD4 and CD8 T cell proliferation in response to peptides. FC dot-plots represent the frequency of activated memory CD4 (first row) and CD8 (second row) T cells in unstimulated or eMMP, tPA-MMP, tPA-MMP-2mut, and tPA-MMP-5mut-stimulated mdPBMC. The profiles are representative of replicates of the FC data obtained from the three steers used in the study.

The flow cytometric assay used in this study was developed and validated in previous studies. It was designed to more accurately identify and phenotype antigen specific CD4 and CD8 T cells proliferating in response to Ag stimulation (22, 30). To develop the assay, a fluorescent vital dye (hydroethidine) was used to identify all cells present in a culture of PBMC stimulated with concanavalin A. Based on side vs. forward light scatter (SSC vs. FSC), activated proliferating cells could be distinguished from unactivated (resting) cells based on an increase in size of activated/stimulated cells. The populations could be distinguished from each other for further analysis by placing electronic gates on the populations and adding an artificial color to them to distinguish the two populations (e.g., coloring unstimulated cells orange and stimulated cells blue). When hydroethidine labeled cells were mixed with a preparation of unstimulated PBMC, the two populations could be distinguished from each other based on fluorescence (hydroethidine labeled cells fluoresce in the red Fl-2 channel). Unstimulated cells in both populations exhibited the same pattern of color coding, distinguishing them from stimulated cells coded with a different color. When the mixed populations were labeled with a monoclonal antibody (mAb) specific for CD45R0, a memory T cell marker, expressed on stimulated and unstimulated cells, unstimulated CD45R0 positive cells in both populations could be distinguished from the stimulated population based on difference in color using FL2 vs. FL1. When the mixed populations of cells were labeled with a mAb only expressed on stimulated cells (e.g., ACT1), unstimulated cells that did not express ACT1 could be distinguished from stimulated cells that expressed ACT1 (30). Further studies demonstrated that Ag specific memory CD4 and CD8 T cells proliferating in response to Ag stimulation *ex vivo*, could be distinguished from unstimulated memory T cells based on the use of selective gating alone to distinguish activated cells based on size and expression of CD45R0 and molecules only expressed on activated T cells. As reported here, a single selective gate was placed on cells displayed in SSC vs. FSC to isolate the population for analysis. A second selective gate was placed on either CD4 or CD8 T cell populations displayed in FSC vs. CD4 or CD8. Both isolated populations were then displayed in FSC vs. CD45R0 for analysis. A box gate was placed on the proportion memory cells showing an increase in size based on FSC (Supplementary Figure 4) (22). This corresponded to the activated cells identified using the initial color coding and gating strategy (30). The proportion of cells present in this gate in preparation of cells from unstimulated cultures were compared with the proportion of cells present in stimulated cultures to determine the increase in the proportion of cells associated with Ag stimulation. Previous studies had documented that these cells were the only cells positive for molecules only expressed on activated cells. Analysis of mdPBMC pulsed with the positive control eMMP showed that the activation and proliferative responses of the memory CD4 and CD8 T cells were consistent with responses obtained in previous experiments using two rounds of stimulation of mdPBMC with antigen-pulsed APC. Comparison of the proliferative responses of CD4 and CD8 T cells elicited by eMMP with responses elicited by tPA-MMP and tPA-MMP-2mut showed that the responses were similar. In contrast, the proliferative response elicited by tPA-MMP-5mut was limited as illustrated with a representative set of flow cytometric profiles obtained from one of three duplicate sets of experiments conducted with one of the three steers used in the study (Figure 6). Therefore, tPA-MMP-5mut was excluded from further analysis.

### Comparison of CTL Activity

**Figure 7:**
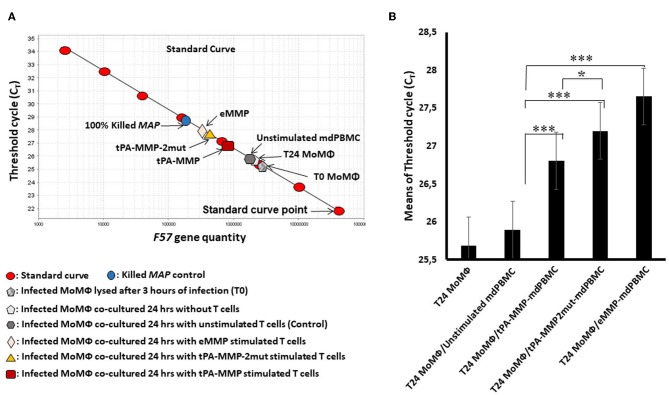
Intracellular *Map* PMA-QPCR viability assay. **(A)** Graphical representation of real-time PCR data for *Map* intracellular killing to determine the C_T_ values measured for amplification of the *Map* F57 target gene. C_T_ values represent average of duplicate preparations of DNA isolated from pure *Map* (8 red oval points), *Map* viability control (blue circle), *Map*-infected MoMΦ lysed after 3 h of infection (gray pentagon), *Map*-infected MoMΦ lysed after 24 h of infection (light gray pentagon), cell mixture lysed after 24 h of co-culturing of *Map*-infected MoMΦ with unstimulated mdPBMC (dark gray hexagon), cell mixture lysed after 24-h co-culture of *Map*-infected MoMΦ with mdPBMC stimulated twice with tPA-MMP (dark red rectangle), tPA-MMP-5mut (gold triangle), or eMMP (pink diamond). **(B)** Summary of three biological replicate experiments performed in duplicate from each of the three steers comparing the killing activity following stimulation with the different MMP peptides relative to controls. *P < 0.05; ***P < 0.001.

As illustrated in Figure 7A, a standard curve was generated with DNA from the single-copy gene, F57, for comparison of the proportion of viable bacteria present in each preparation of *Map* isolated from each cell preparation. Because blocking of F57 DNA by PMA is incomplete, a second control preparation was made from macrophages infected with a known concentration of live and dead bacteria to establish where the concentrations of live and dead bacteria will fall on the standard curve. This control was also used to show the sensitivity of the assay to determine the relative proportion of live bacteria present in each culture of infected target cells at the time of DNA isolation. Comparison of live bacteria isolated from control-infected macrophages immediately after infection with live bacteria isolated at 24 h after infection (identical to the time infected macrophages were incubated with unstimulated and antigen stimulated mdPBMC) showed that there was limited loss of live bacteria during the 24-h incubation period. There was also limited loss of live bacteria in infected macrophage preparations incubated with unstimulated mdPBMC. In contrast, there was a clear loss of live bacteria in preparations of infected macrophages incubated with eMMP, tPA-MMP, and tPA-MMP-2mut. Comparison of the CTL activity of CD8 T cells from the three steers that proliferated in response to stimulation with eMMP, tPA-MMP, and tPA-MMP-2mut showed that the CTL activity of CD8 T cells stimulated with tPA-MMP-2mut was consistently better than the CTL activity of CD8 T cells stimulated with tPA-MMP (Figure 7B). However, both CTL responses were slightly less than the response elicited with eMMP.

## Discussion

Efforts to develop vaccines against mycobacterial pathogens have been impeded by the lack of understanding the mechanisms used by the pathogens to establish a persistent infection. This occurs concurrently with the development of humoral and cell-mediated responses to the mycobacteria that are able to delay development of clinical disease. In humans, the immune response that develops against *Mtb* is sufficient to maintain immune control for a lifetime in most exposed subjects. Clinical disease occurs in ~10% of individuals persistently infected with *Mtb*, and is associated with impairment of immune function (35). This may also occur in response to infection with *Map* in humans (3–5, 36). Studies in cattle have shown that exposure leads to development of a latency that varies in duration, with a breakdown in protective immunity occurring two or more years post exposure. The immune response during the early stages of infection is resilient and not readily disrupted by use of immunosuppressive agents like dexamethasone or depletion of CD4 T cells at the time exposure (37). The response is sufficient to suppress bacterial replication to the extent that it is difficult to detect the presence of bacteria in any tissue (17). Eventually, however, for yet to be explained reasons, protective immunity is disrupted and clinical disease develops.

Of importance to the development of a vaccine against *Map* and other mycobacterial pathogens, this evasion tactic is lost when *relA* is disrupted. This was first demonstrated by Dahl et al. using a mouse model with a *relA Mtb* deletion mutant. The mutant was unable to establish a persistent infection (20). Studies with *Map* revealed that deletion of *relA* had the same effect. Infection with the mutant was cleared (38). Development of a flow cytometric assay and a bacterium viability assay to replace the cfu assay facilitated characterization of the phenotype and functional activity of CD4 and CD8 T cells proliferating during the recall response following stimulation with the *Map relA* mutant. Analysis revealed that clearance was attributable to development of CD8 CTL with the ability to kill intracellular bacteria (22). Analysis of the response led to the discovery that a 35-kDa membrane-associated peptide, MMP, is the target of the CTL response (22). Vaccination with a replicate of the peptide expressed in *E. coli*, eMMP, elicited a CTL response equivalent to the response elicited by the Δ*Map/relA* mutant, suggesting that it might be possible to develop a peptide-based vaccine for Ptb. Analysis of the immune response to the peptide revealed a previously unrecognized feature of the response essential for eliciting CTL. Development of CD8 CTL only occurred if CD4 and CD8 T cells recognize their respective epitopes presented by APC at the same time. Using the *ex vivo* test platform, examination of the CTL response to stimulation with APC pulsed with MMP revealed that the CTL response was blocked in the presence of antibody to either MHC I or MHC II molecules (in review). These results showed that development of a peptide-based vaccine with eMMP would require retention of epitopes in the peptide that would be processed and presented in the context of MHC I and II molecules. This is a new salient observation that may be relevant for development of peptide-based vaccines not only for *Map* but also for other pathogens.

The first choice for a vector-based vaccine was the use of nanoparticles. Trial experiments with a well-characterized nanoparticle revealed that an equivalent CTL response to MMP could be elicited *ex vivo* with MMP alone or incorporated in a nanoparticle vector (24). Unfortunately, attempts to standardize a method of production of a nanoparticle vectored MMP vaccine for field use demonstrated that it was not possible to make a product with reliable activity, indicating that other approaches needed to be explored for the development of an MMP-based vaccine.

Extensive studies have been conducted to develop and test viral vectors for delivery of vaccines [reviewed in (39, 40)]. There has been one attempt to develop a virus-vectored vaccine for *Map* (14). Methods were unavailable to analyze the functional activity of the expressed products. To explore the potential of developing a virus-vectored vaccine for MMP, information from ongoing studies revealed that the first step would require development of an expression cassette for insertion and testing of available viral vectors for delivery of MMP as a vaccine. Of critical importance, studies demonstrated any modifications of the native sequence of the gene encoding MMP must not alter the immunogenic properties of the expressed peptide. Antigen processing of any modified expressed copy of MMP by APC must lead to expression of the same epitopes in the context of MHC I and II molecules (in review). Although extensive information is available on the use of bacteria to express mammalian proteins in bacteria, information is still limited on expression of mammalian proteins through bacterial secretory pathways. The main obstacle for both viral and DNA vector-based vaccines delivering bacterial antigens is maintenance of antigen authenticity when expressed by mammalian cells. In contrast to mammalian cells, proteins expressed by bacteria do not undergo post-translational modification. Consequentially, modification for expression in mammalian cells could negatively impact peptide immunogenicity. Therefore, the design of a cassette for use in expression of bacterial proteins in mammalian expression vectors needs to be taken into consideration. As reported here, the first step in accomplishing this objective was conducted on the premise that modification of the native gene sequence encoding MMP for expression in mammalian cells, specifically human derived cell lines, would yield an expressed product with the same immunogenic activity as the native molecule expressed by MAP2121c. Proof of this premise would not be known until a modified sequence was developed and used to express a product for analysis of its immunogenic properties.

*In silico* analysis of the MMP sequence was conducted to predict the protein topology when expressed in mammalian cells. Although previous studies provided data indicating MMP is localized on the surface in complex with another peptide (23, 29), it was surprising to find that the peptide did not contain a canonical signal peptide, transmembrane domain, or a GPI anchor to sort and link MMP to the bacterial cell surface. In fact, the *in silico* analysis predicted localization of MMP in the cytoplasm of transfected or transduced mammalian cells. However, the finding is not unprecedented. Similar observations have been obtained in the study of other mycobacteria (41, 42).

Protein expression levels are closely related to genetic sequences, which are often similar but never identical between species of organisms. Comparative studies have revealed the use of two to six different synonymous DNA triplets to encode the same amino acid with some of the codons used more frequently than others, a phenomenon defined as codon usage bias, which is directly related to gene expression efficiency. This must be considered when modifying codon usage for expression of recombinant proteins by different species, in order to obviate codon usage differences and maximize efficiency of expression (43–45). In this study, MMP ORF codon usage was modified with the aid of the Java Codon Adaptation Tool (JCat) for expression of MMP in mammalian cells. The adapted MMP ORF sequence was modified further to include a mammalian signal peptide sequence to facilitate efficient intracellular expression and extracellular secretion. A third-generation, replication-incompetent pEF1α-tPA-MMP-CMV-EGFP:T2A:Puro lentiviral vector was used to generate a recombinant lentivirus tPA-MMP expression cassette. Initial use of the cassette demonstrated that it could be used to successfully transduce different cell lines and primary cell cultures (data not shown). Particularly, we generated stable MMP lentivirus transduced human cell lines constitutively secreting tPA-MMP, tPA-MMP-2mut, and tPA-MMP-5mut for *ex vivo* immunization studies. We took advantage of the ability to culture the transduced cell lines in serum-free medium to produce supernatants containing the three variants of tPA-MMP almost free of non-specific proteins for use in analysis of its immunogenic activity.

SDS-PAGE and Western blot analysis of the secreted products revealed that they exhibited a slightly higher molecular weight than the expected ~35 kDa of the native MMP. *In silico* examination of the modified amino acid sequence of tPA-MMP using different software programs revealed that some amino acids that might be glycosylated, explaining the unexpected difference in the pattern of electrophoretic migration. To explore this possibility, asparagine residues in tPA-MMP predicted to be potential sites for N-glycosylation were substituted with glutamine, a structurally similar amino acid that is not a target for glycosylation or fucosilation. In addition, supernatants containing the tPA-MMP variants were digested with PNGase F, a deglycosylation enzyme. Both manipulations of the modified tPA-MMP confirmed that N-linked glycosylation occurred when the expression vector was transfected or transduced into mammalian cells. Both manipulations yielded a product with the expected molecular weight of native MMP as detected by its pattern of migration in SDS-PAGE.

Following physical characterization of the modified forms of tPA-MMP, it was possible to finally determine if the modifications of MMP for expression in a mammalian cell altered the capacity of MMP to elicit CTL activity essential for use of MMP in a peptide-based vaccine. As reported, the use of the *ex vivo* platform and bacterium viability assay facilitated analysis of the proliferative and functional activity of tPA-MMP and the mutants in comparison with MMP expressed in *E. coli* (eMMP). Comparison of the proliferative responses revealed that stimulation with APC pulsed with eMMP, tPA-MMP, and the tPA-MMP-2mut yielded similar proliferative responses. Stimulation with tPA-MMP-5mut yielded minimal proliferation, suggesting that the exchange of asparagine with glutamine affected the immunogenic characteristics of the peptide. Based on this finding, tPA-MMP-5mut was excluded from further testing. Additional comparisons of the capacity of the expressed, modified tPA-MMPs revealed modest difference in the capacity of tPA-MMP and tPA-MMP-2mut to elicit CTL response. The findings suggest that both tPA-MMP and tPA-MMP-2mut are suitable candidates for use in a virus-vectored vaccine for *Map*. Further studies are now warranted to use the shuttle vector to develop virus vectors containing tPA-MMP or tPA-MMP-2mut to test for efficacy.

## Data Availability Statement

All datasets generated for this study are included in the article/Supplementary Material.

## Ethics Statement

All protocols were reviewed and approved by the Washington State University Institutional Animal Care and Use Committee (ASAFs 3360 and 04883).

## Author Contributions

GD conceived the experiments. VF, AM, GA, GT, FM, LR, ME, and GD performed the experiments. JB, K-TP, VH, and SC contributed with reagents. GD, LF, and WD analyzed the data. LF edited the paper. GD and WD wrote the paper. VF and AM shared equally in the conduct of the study. All authors participated in the review of the present version of the manuscript.

### Conflict of Interest

The authors declare that the research was conducted in the absence of any commercial or financial relationships that could be construed as a potential conflict of interest.

## Abbreviations

APC: antigen-presenting cells
pA: bovine growth hormone polyadenylation signal
CBS: calf bovine serum
CTL: cytotoxic T cells
cRPMI: complete culture medium
cDC: conventional dendritic cells
DMSO: dimethyl sulfoxide
EMEM: Eagle's minimal essential medium
EDTA: ethylenediaminetetraacetic acid
FBS: fetal bovine serum
GFP: green fluorescence protein
CMV: human cytomegalovirus
EF1α: human elongation factor 1 alpha promoter
HEK: human embryo kidney
HKB-11: human kidney/B cell hybrid
tPA: human tissue plasminogen activator signal peptide
*Map*: *Mycobacterium avium* subsp. *paratuberculosis*
eMMP: MMP expressed in *E. coli*
MoDC: monocyte-derived dendritic cells
mdPBMC: monocytes depleted PBMC
MoMΦ: monocyte derived macrophages
GlcNAc: N-acetyl-D-glucosamine
NP: nanoparticles
Ptb: paratuberculosis
PBMC: peripheral blood mononuclear cells
PBS: phosphate buffered saline
PEI: polyethylenimine
PMA-QPCR: propidium monoazide quantitative PCR
qRT-PCR: quantitative real-time PCR
MMP: 35 kDa membrane peptide.
