# Supplementary material for: Capacity to Elicit Cytotoxic CD8 T Cell Activity Against Mycobacterium avium subsp. paratuberculosis Is Retained in a Vaccine Candidate 35 kDa Peptide Modified for Expression in Mammalian Cells
Source: Front Immunol. 2019 Dec 11;10:2859. doi: 10.3389/fimmu.2019.02859 (PMC6917596; doi:10.3389/fimmu.2019.02859)
Supplement: Supplementary file 1 [file Data_Sheet_1.PDF]

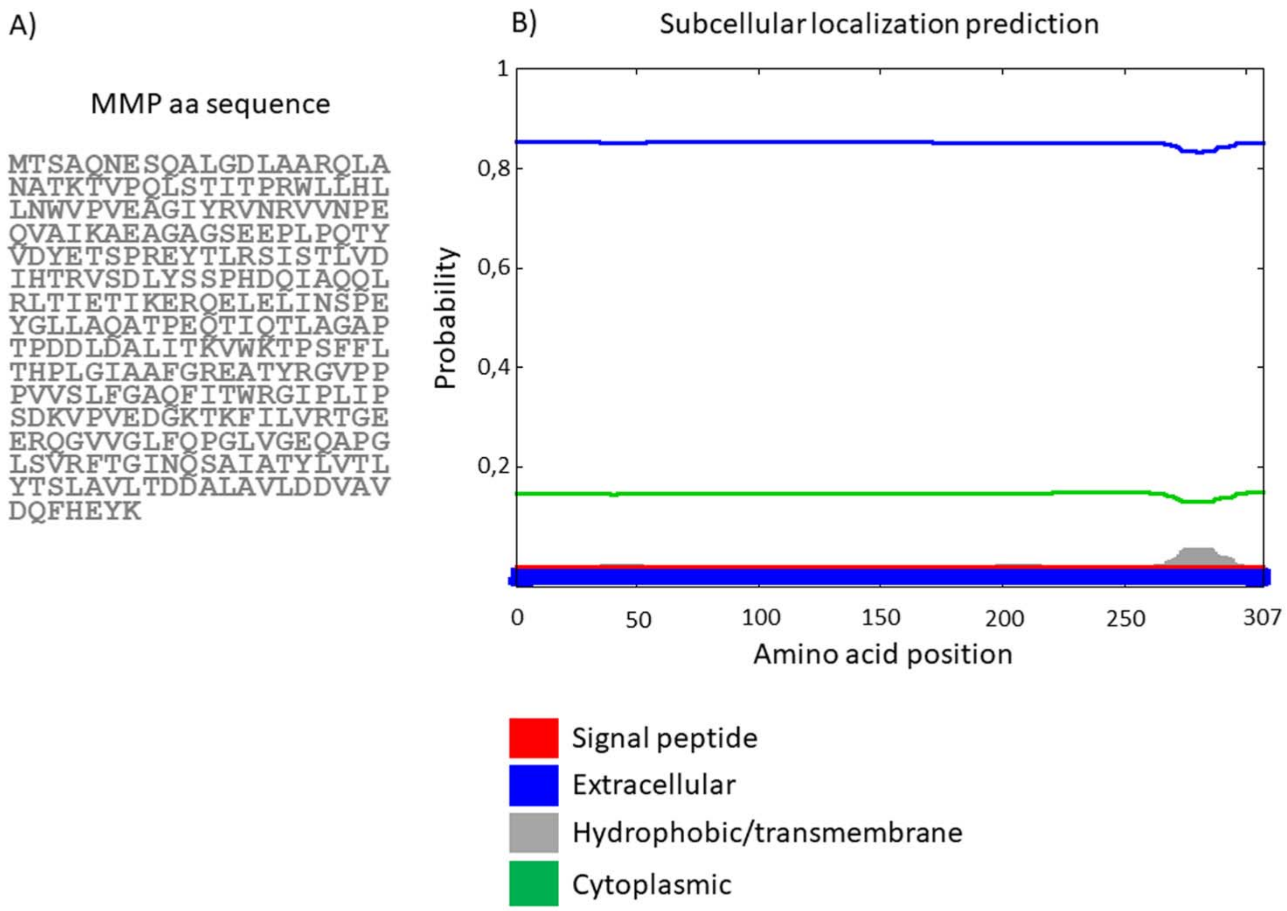

Supplementary Figure 1. A) Amino acid composition and **B)** server output prediction of transmembrane topology and signal peptides from the amino acid sequence of MMP protein. None of them reach the top score of probability (1).

A)

MMP ORF sequence

```
ATGACGTCGGCTCAAAATGAGTCTCAAGCACTTGGTGATCTGGCTGCCAG   50
GCAACTCGCCAACGCAACCAAGACCGTCCCCCAGCTCTCGACGATCACGC   100
CGCGCTGGCTGCTGCACCTGCTGAACTGGGTTCCGGTGGAGGCGGGCATC   150
TACCGGGTGAACCGGGTGGTCAATCCCGAGCAGGTCGCCATCAAGGCCGA   200
GGCCGGCGCCGGCAGTGAAGAGCCGCTACCGCAGACCTATGTGGACTACG   250
AGACCAGCCCGCGCGAGTACACGCTGCGCAGCATTTCCACGCTGGTCGAC   300
ATCCACACCCGGGTCTCCGACCTGTACTCGAGCCCGCACGATCAGATCGC   350
CCAGCAGCTGCGGCTGACCATCGAGACCATCAAGGAGCGCCAGGAGCTGG   400
AGCTGATCAACAGCCCCGAGTATGGGCTGCTGGCCCAGGCGACGCCGGAG   450
CAGACGATCCAGACGCTGGCCGGGGCTCCCACGCCCGACGACCTCGACGC   500
GCTGATCACCAAGGTGTGGAAGACGCCCAGTTTCTTCCTGACCCACCCGC   550
TGGGCATCGCGGCGTTCGGGCGCGAGGCCACCTACCGGGGGGTGCCGCCG   600
CCGGTGGTGAGCCTGTTCGGCGCCCAGTTCATCACCTGGCGCGGTATTCC   650
GCTGATCCCGTCGGACAAGGTGCCGGTGGAGGACGGCAAGACGAAGTTCA   700
TCCTGGTCCGCACCGGCGAGGAACGTCAGGGCGTCGTCGGGCTGTTCCAG   750
CCCGGCCTGGTCGGGGAGCAGGCGCCGGGGCTGTCGGTGCGGTTCACCGG   800
CATCAACCAGTCGGCGATCGCGACCTACCTGGTCACGCTGTACACCTCCC   850
TGGCCGTCCTGACCGATGACGCGCTCGCCGTGCTCGACGACGTCGCGGTG   900
GATCAGTTCCATGAGTACAAGTGA
```

B)

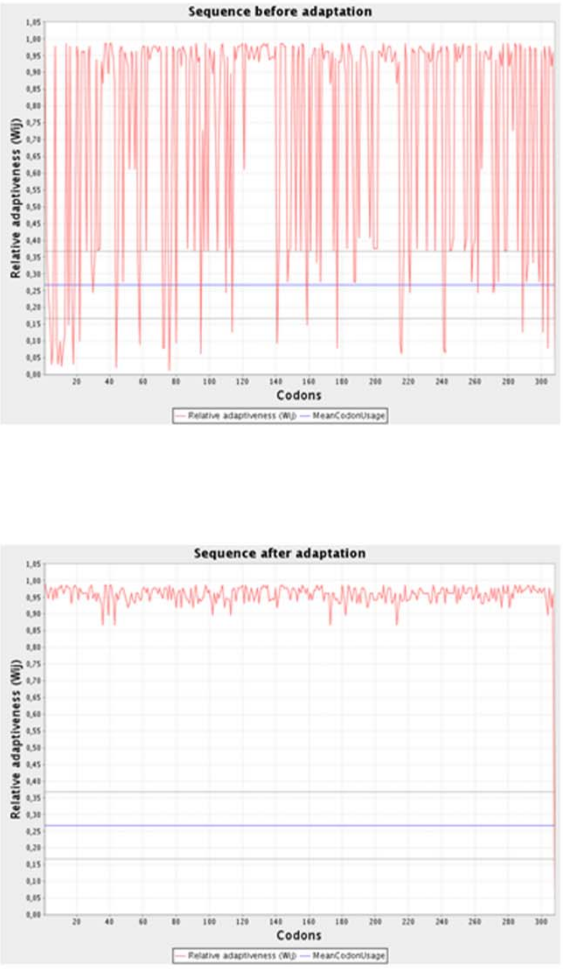

C)

MMP ORF sequence Human adapted

```
ATGACCAGCGCCCAGAACGAGAGCCAGGCCCTGGGCGACCTGGCCGCCCG   50
CCAGCTGGCCAACGCCACCAAGACCGTGCCCCAGCTGAGCACCATCACCC   100
CCCGCTGGCTGCTGCACCTGCTGAACTGGGTGCCCGTGGAGGCCGGCATC   150
TACCGCGTGAACCGCGTGGTGAACCCCGAGCAGGTGGCCATCAAGGCCGA   200
GGCCGGCGCCGGCAGCGAGGAGCCCCTGCCCCAGACCTACGTGGACTACG   250
AGACCAGCCCCCGCGAGTACACCCTGCGCAGCATCAGCACCCTGGTGGAC   300
ATCCACACCCGCGTGAGCGACCTGTACAGCAGCCCCCACGACCAGATCGC   350
CCAGCAGCTGCGCCTGACCATCGAGACCATCAAGGAGCGCCAGGAGCTGG   400
AGCTGATCAACAGCCCCGAGTACGGCCTGCTGGCCCAGGCCACCCCCGAG   450
CAGACCATCCAGACCCTGGCCGGCGCCCCCACCCCCGACGACCTGGACGC   500
CCTGATCACCAAGGTGTGGAAGACCCCCAGCTTCTTCCTGACCCACCCCC   550
TGGGCATCGCCGCCTTCGGCCGCGAGGCCACCTACCGCGGCGTGCCCCCC   600
CCCGTGGTGAGCCTGTTCGGCGCCCAGTTCATCACCTGGCGCGGCATCCC   650
CCTGATCCCCAGCGACAAGGTGCCCGTGGAGGACGGCAAGACCAAGTTCA   700
TCCTGGTGCGCACCGGCGAGGAGCGCCAGGGCGTGGTGGGCCTGTTCCAG   750
CCCGGCCTGGTGGGCGAGCAGGCCCCCGGCCTGAGCGTGCGCTTCACCGG   800
CATCAACCAGAGCGCCATCGCCACCTACCTGGTGACCCTGTACACCAGCC   850
TGGCCGTGCTGACCGACGACGCCCTGGCCGTGCTGGACGACGTGGCCGTG   900
GACCAGTTCCACGAGTACAAGTAA
```

D)

Supplementary Figure 2. Human codon usage unadapted **(A)** and adapted **(C)** MMP sequence and their GC content respectively **(B and D)**. The red line shows the Codon Usage for each codon resent in the gene. The blue line the mean codon usage in the unadapted sequence. The grey lines above and below the blue line mark the standard deviation for this mean codon usage in the selected organism. In the adapted sequence, all codons were shifted to the value 1.0, which is the maximum value for codons.

MMP-2mut

```
atgaccagcgcccagcaagagagccaggccctgggcgacctggccgcccgccagctggcc
 M   T   S   A   Q  1Q   E   S   Q   A   L   G   D   L   A   A   R   Q   L   A
caagccaccaagaccgtgccccagctgagcaccatcacccccgctggctgctgcacctg
2Q   A   T   K   T   V   P   Q   L   S   T   I   T   P   R   W   L   L   H   L
ctgaactgggtgcccgtggaggccggcatctaccgcgtgaaccgcgtggtgaaccccgag
 L   N   W   V   P   V   E   A   G   I   Y   R   V   N   R   V   V   N   P   E
caggtggccatcaaggccgaggccggcgccggcagcgaggagcccctgccccagacctac
 Q   V   A   I   K   A   E   A   G   A   G   S   E   E   P   L   P   Q   T   Y
gtggactacgagaccagcccccgcgagtacaccctgcgcagcatcagcaccctggtggac
 V   D   Y   E   T   S   P   R   E   Y   T   L   R   S   I   S   T   L   V   D
atccacacccgcgtgagcgacctgtacagcagcccccacgaccagatcgcccagcagctg
 I   H   T   R   V   S   D   L   Y   S   S   P   H   D   Q   I   A   Q   Q   L
cgcctgaccatcgagaccatcaaggagcgccaggagctggagctgatcaacagccccgag
 R   L   T   I   E   T   I   K   E   R   Q   E   L   E   L   I   N   S   P   E
tacggcctgctggcccaggccacccccgagcagcaaatccagcaactggccggcgccccc
 Y   G   L   L   A   Q   A   T   P   E   Q   T   I   Q   T   L   A   G   A   P
acccccgacgacctggacgccctgatcaccaaggtgtggaagacccccagcttcttcctg
 T   P   D   D   L   D   A   L   I   T   K   V   W   K   T   P   S   F   F   L
acccacccccctgggcatcgccgccttcggccgcgaggccacctaccgcggcgtgcccccc
 T   H   P   L   G   I   A   A   F   G   R   E   A   T   Y   R   G   V   P   P
cccgtggtgagcctgttcggcgcccagttcatcacctggcgcggcatcccccctgatcccc
 P   V   V   S   L   F   G   A   Q   F   I   T   W   R   G   I   P   L   I   P
agcgacaaggtgcccgtggaggacggcaagaccaagttcatcctggtgcgcaccggcgag
 S   D   K   V   P   V   E   D   G   K   T   K   F   I   L   V   R   T   G   E
gagcgccagggcgtggtgggcctgttccagcccggcctggtgggcgagcaggcccccggc
 E   R   Q   G   V   V   G   L   F   Q   P   G   L   V   G   E   Q   A   P   G
ctgagcgtgcgcttcaccggcatccaacagagcgccatcgccacctacctggtgaccctg
 L   S   V   R   F   T   G   I   N   Q   S   A   I   A   T   Y   L   V   T   L
tacaccagcctggccgtgctgaccgacgacgccctggccgtgctggacgacgtggccgtg
 Y   T   S   L   A   V   L   T   D   D   A   L   A   V   L   D   D   V   A   V
gaccagttccacgagtacaagtaa
 D   Q   F   H   E   Y   K   -
```

N to Q (only on position 1 and 2)

Supplementary Figure. 3. Mutated MMP protein sequences (MMP-2mut), where the predicted N-linked Asparagines residues were substituted with Glutamine (Q, red) only on the position 1 and 2.

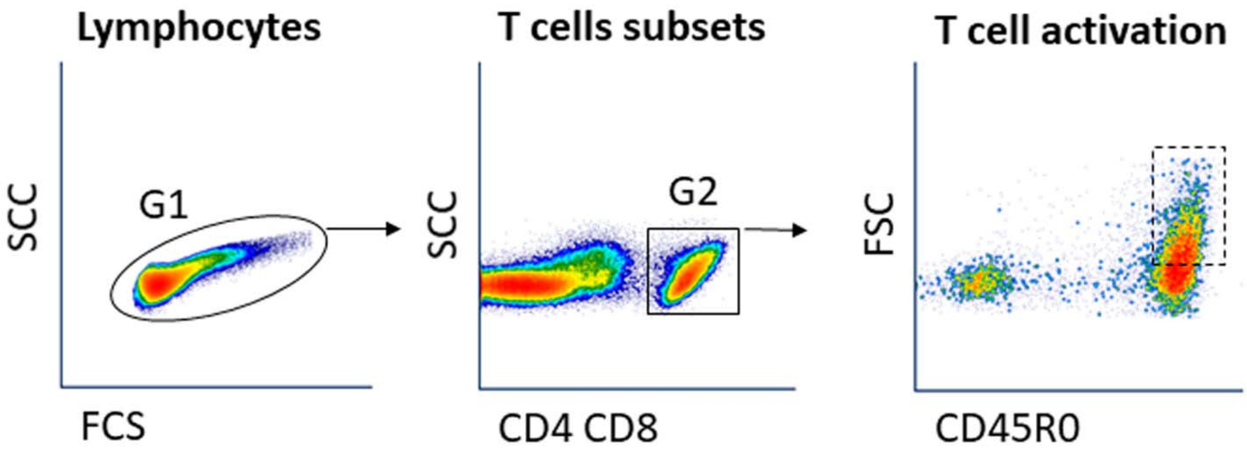

Supplementary Figure 4. Flow cytometric gating strategy for analysis of the CD4 and CD8 T cell proliferative response. Side versus forward scatter (SSC vs FSC) gating was used to record small and large activated lymphocytes (G1) based on their scatter properties, followed by a single-cell gating (FSC-H vs FSC-W) to exclude doublets (G2). G2 was used in single-parameter analysis to further identify the distinct T cell populations including CD4 and CD8 T cells. Additional gates on CD4 or CD8 cells (G3) were used in two-parameter analysis (FSC vs CD45R0 (memory marker)) to determine the activated memory cells used in the analysis (boxed rectangle) within each cell population.
